# The Role of a Radiology Electronic Notification System in the Emergency Department Setting and Its Impact on Patient Care

**DOI:** 10.14740/jocmr1821w

**Published:** 2014-05-22

**Authors:** Lena A. Omar, Richard V. King, John Pease, Hythem A. Omar

**Affiliations:** a Department of Radiology, UT Southwestern Medical Center, 5323 Harry Hines Boulevard, MC 8896, Dallas, TX 75390, USA; b Health Care Sciences/Emergency Medicine Education, UT Southwestern Medical Center, 5323 Harry Hines Boulevard, MC 9134, Dallas, TX 75390, USA; c Emergency Medicine, Department of Surgery, Quality and Safety of Emergency Services at Parkland Health & Hospital System, UT Southwestern Medical Center, 5323 Harry Hines Boulevard, MC 8579, Dallas, TX 75390, USA

**Keywords:** Emergency department, Radiology electronic notification system, Patient care

## Abstract

**Background:**

To determine the impact of a radiology electronic notification system (ENS) on emergency department (ED) patient care.

**Materials and Methods:**

A retrospective review of de-identified patient data for a 2-year period (1 year prior to and 1 year following ENS implementation) was approved by the hospital’s institutional review board. The effect of a radiology ENS on ED patient care was investigated by analyzing the intervals between completion of a chest radiograph and the times antibiotics were ordered/administered on patients presenting with symptoms of community acquired pneumonia (CAP). The square root transformation of the means was analyzed with an ANOVA model to determine statistical significance.

**Results:**

During the 24-month study protocol, 1,341 patients who were evaluated in the ED met the study eligibility criteria. The least square estimates of the mean times from when the chest radiograph was completed to when antibiotics were ordered prior to and after the implementation of the ENS were 89 and 107 minutes, respectively (P < 0.01). The least square estimates of the mean times from when the chest radiograph was completed to when antibiotics were administered prior to and after the implementation of the ENS were 115 and 132 minutes, respectively (P = 0.02).

**Conclusion:**

The implementation of a radiology ENS does have advantages for the radiologist in streamlining the communication and documentation processes but may negatively impact time to treatment and thus patient care.

## Introduction

According to the Centers for Disease Control and Prevention, 1.1 million people were discharged from US hospitals with a diagnosis of pneumonia in 2009. That same year, over 50,000 people died from pneumonia [1]. Community acquired pneumonia (CAP) affects individuals who have not had recent exposure to hospitals or health care facilities such as nursing homes or dialysis units. *Streptococcus pneumoniae*, *Haemophilus influenzae* and *Moraxella catarrhalis* are responsible for approximately 85% of cases of CAP [2]. Without timely and appropriate treatment, CAP can carry a poor prognosis leading to sepsis, shock and even death. The Joint Commission on Accreditation of Healthcare Organizations has established core measures concerning preventative care and treatment for, among other diseases, CAP including timely antibiotic administration. Past studies have demonstrated that patients with comorbidities, such as diabetes mellitus, experience increased mortality when initial antibiotics in the emergency department (ED) setting are delayed [3]. Furthermore, other factors including advanced age and multilobar involvement have also proved to be poor prognostic indicators [2]. As ED patient population complexity and volume have increased, the time to first antibiotics for patients with CAP has also increased beyond quality measure guidelines [4].

Many hospitals and institutions utilize an electronic medical notification system to alert providers of critical test results. In the outpatient setting, it has been shown that automatic notification of abnormal laboratory results has not necessarily resulted in improved timely patient follow-up [5]. However, Eisenberg et al concluded that a radiology electronic messaging system was effective in communicating non-emergent results to referring physicians in an efficient and inexpensive manner (albeit without a control group) [6]. To the authors’ knowledge, the effects of a radiology electronic notification system (ENS) in the emergency setting with a control assessment pre-implementation have not been previously investigated.

For this study, de-identified data were examined from all CAP patients presenting to the hospital’s ED over the year before and year after radiology’s implementation of an ENS. The goal was to determine the impact of a radiology ENS on ED patient care. The implementation of the ENS was part of a complex quality improvement process that helped raise awareness of CAP and core measures, thus leading to a much improved institutional dashboard for CAP. Moreover, this was a collaborative multidisciplinary effort bringing various departments together under the umbrella of quality improvement.

## Materials and Methods

### Study subjects

This hospital’s institutional review board approved this retrospective study. HIPAA compliant de-identified data were analyzed representing 1,341 patients presenting to the ED from July 2009 through July 2011. Six hundred sixty-three patients were seen in the year preceding the radiology department’s July 1, 2010 ENS implementation and 678 patients in the year following implementation. Both groups comprise a consecutive series of patients meeting the inclusion criteria. Inclusion criteria for the study required that subjects were ED patients with clinical suspicion of CAP based on history and physical exam (fever, productive cough, chills) along with radiographic evidence of new or focal infiltrate on chest imaging obtained as part of their ED diagnostic workup. Patients with radiographic evidence of chronic infiltrates and diffuse interstitial opacities were excluded. Finally all chest imaging had to be interpreted by a board certified or in-training radiologist as being compatible with a pneumonic process.

### Study protocol

**Figure 1:**
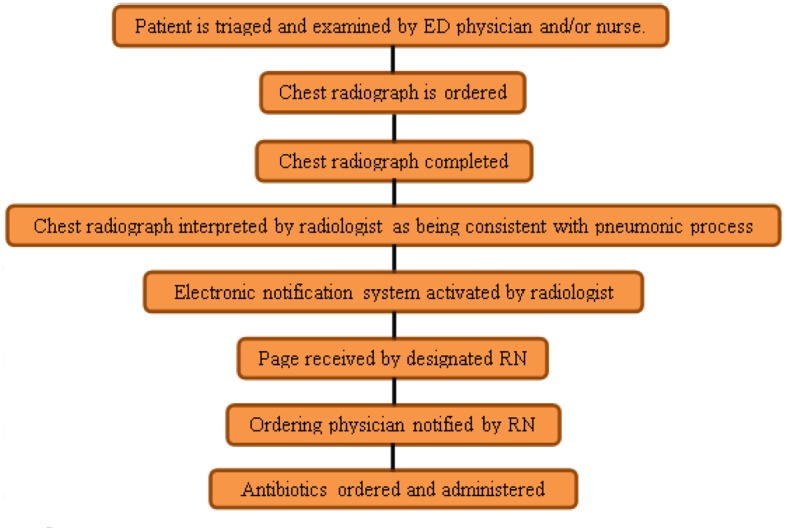
Workup protocol employing a radiology ENS for ED patients suspected of having CAP.

All patients were initially triaged by the ED as to the acuity of their presenting complaints and symptoms. Patients underwent a clinical examination by an ED staff physician, resident and/or nurse, including appropriate clinical history and physical examination. If the patient met the inclusion criteria, a chest radiograph was initially ordered along with other laboratory data. If the chest radiograph demonstrated findings consistent with a pneumonic process, then the interpreting radiologist would activate the ENS immediately prior to signing the report. As per protocol, a designated ED registered nurse would receive the message via his/her pager and would then alert the treating doctor as to the findings (Fig. 1). Messages were sent to a single pager (designated as the CAP pager) to avoid the confusion/difficulty of reaching physicians who may have not examined the patient yet or who were off ED duty at the time of image interpretation.

Two parameters were measured: the time interval between chest radiograph completion and antibiotics ordered, and the time interval between chest radiograph completion and antibiotics administered. Data were obtained for both subsets of patients (pre- and post-implementation of the ENS).

### Statistical analysis

**Figure 2:**
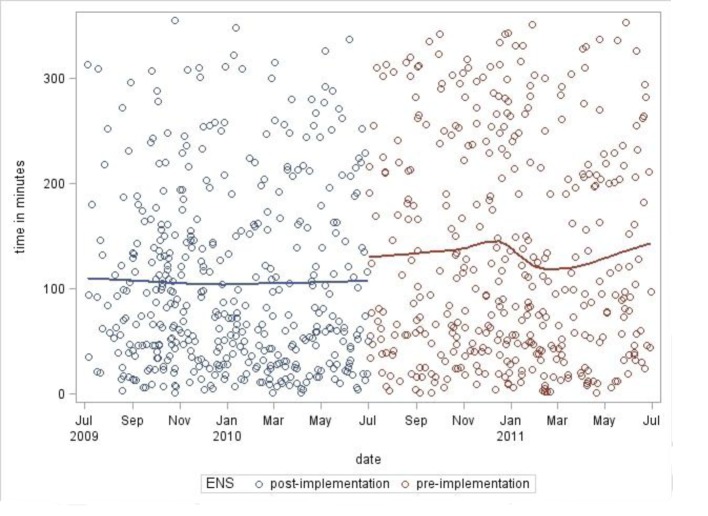
Scatterplot of time between chest radiograph completed to antibiotics ordered.

**Figure 3:**
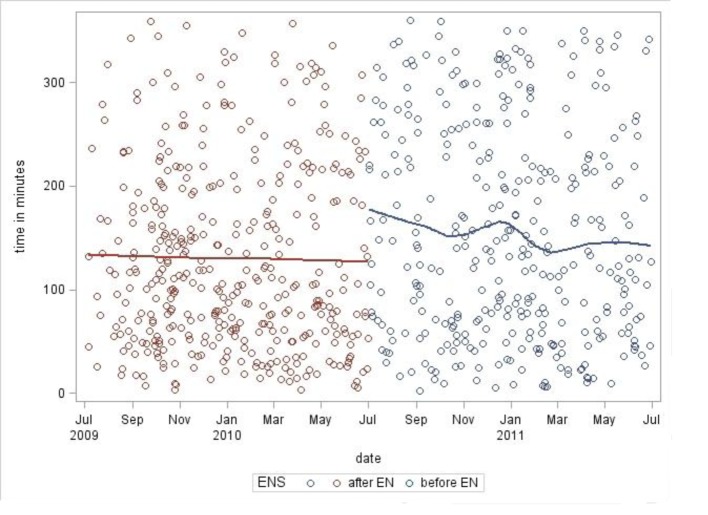
Scatterplot of time between chest radiograph completed to antibiotics administered.

**Figure 4:**
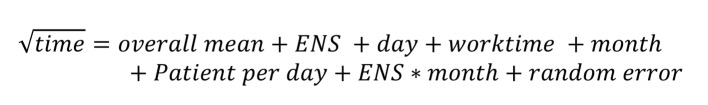
ANOVA model where ENS indicates pre- or post-implementation of the ENS device and ENS * month is the interaction between ENS and month.

Data were entered into a statistical software program and analyzed. Scatter plots for each group of data were performed analyzing the times between chest radiograph completion and antibiotics ordered/administered as a function of calendar months of the year (Fig. 2, 3). Solid lines show the trend of the data. Square root transformation of the means was performed to compensate for the skewness of the raw data. An ANOVA model was employed to account for various factors including time of day at presentation, day of the week, month of the year, number of ED patients per day and random error (Fig. 4).

This hospital’s institutional review board approved this retrospective study.

## Results

During the 24-month study protocol, 1,341 patients were evaluated in the ED that met the study eligibility criteria.

The least square estimate of the mean time from when the chest radiograph was completed to the time when antibiotics were first ordered prior to the implementation of the ENS was 88.67 min (95% CI: 73.11 - 105.71). The least square estimate of the mean time post-implementation was 107.19 min (95% CI: 90.02 - 125.85). Based on the ANOVA, results were statistically significant with P < 0.01.

The least square estimate of the mean time from when the chest radiograph was completed to when antibiotics were administered prior to the implementation of the ENS was 115.42 min (95% CI: 101.91 - 129.77). After implementation, least square estimate of the mean time was 132.28 min (95% CI: 118.44 - 146.88). Based on the ANOVA, results were also statistically significant (P = 0.02).

Scatter plot analyses showed no obvious increasing or decreasing trend in both the pre- and post-ENS implementation groups as a function of linear time.

## Discussion

Appropriate and timely antibiotics are important standard treatment for patients with CAP and are necessary to avoid more serious illness requiring hospitalization and even death. One study found that CAP carries a mortality rate between 8% and 15% in hospitalized patients [7]. The data concerning the effectiveness of antibiotic timing are controversial in the current literature. While Simonetti et al reported no association between early antibiotics and decreased 30-day mortality in patients with CAP, Houck et al showed a relationship between early antibiotic administration and decreased mortality, length of hospital stay and decreased health care costs [8]. In any regard, the authors’ goal was to determine the effect of a radiology department’s ENS on ED patient care. Such a system was established as a means to streamline the communication process as well as provide documentation of communication of critical results.

Past studies have shown that time to follow-up of abnormal imaging results has not always improved after the implementation of an ENS and thus patient care has not necessarily benefited, at least in the outpatient setting [9]. However, Abujudeh et al found that an email alert system relaying important but non-emergent imaging findings has a benefit in the outpatient setting by easing communication between radiologists and ordering physicians in an efficient, traceable manner [10]. Another study by Horri et al revealed that an ENS integrated into picture archiving and communication systems shortened ED physicians’ time in viewing important images and reports [11]. Our results indicate that after the implementation of the ENS, times to antibiotic ordering and administration increased for ED patients with suspected CAP. Results were statistically significant.

In our current model, upon interpreting a chest radiograph consistent with CAP, the radiologist activates the ENS sending an instant message to the designated CAP ED pager, which is carried by the ED charge nurse. This process is captured electronically and documented in the final report. The ED charge nurse then alerts the treating physician. Prior to the ENS, the interpreting radiologist would page the treating physician directly, communicate the results and document the process in his/her report.

While the ENS has taken the burden off the radiologist in terms of communicating with the appropriate ED physician, it has introduced new processes with their own limitations. One possible explanation for our study’s results is the introduction of an intermediary between the radiologist and treating doctor, a necessity given the complexity of ED practitioner services. After receiving the electronic notification, the additional time spent by the charge nurse interrogating the medical record, identifying, finding and notifying the treating physicians may contribute to the prolonged times noted in the study. Moreover, maintaining the CAP pager is only one of many responsibilities undertaken by the ED charge nurse. Other factors that may contribute include year-over-year increased patient volumes thereby taxing ED and physician resources. Results may also be skewed if the CAP pager itself was nonfunctional for a period of time (as may happen if the battery dies without timely attention).

In general, the implementation of any new system requires time to fully implement as people learn the new approach; in some cases, there may be an initial dip in productivity for a period of time. Such conditions may have limited the researchers’ ability to measure the impact of the new approach. However, scatter plot analysis revealed no obvious increasing or decreasing trends as a function of time (Fig. 2, 3). Additionally, our study population is limited to ED patients; a controlled study involving a more comprehensive patient population (inpatients and outpatients) would be ideal. However, having a control assessment in the form of directly comparable data sets pre- and post-ENS implementation is a study strength.

In conclusion, while an ENS does have advantages for the radiologist in streamlining the communication and documentation processes, it negatively impacted patient care by contributing to prolonged time to antibiotic ordering and administration in ED patients with CAP. While further investigation into the efficacy of our ENS takes place, procedures have been implemented to address this study’s findings. These efforts emphasize the importance of a multidisciplinary approach by including the clinical pharmacist on the receiving end of the CAP pager in addition to the ED charge nurse. Throughput efficiencies and other staffing related issues have been addressed ultimately decreasing door to provider times. Furthermore, this study has raised the general awareness as to the need for prompt attention when an electronic notification is received. Subsequently there was a complete streamlining of the “critical value” reporting process. Further investigation with broader patient populations is necessary to fully characterize the ENS’s potential.

## References

[1] www.cdc.gov.

[2] Burke CA, Bronze M Community-Acquired Pneumonia. emedicine.medscape.com.

[3] Bader MS, Abouchehade KA, Yi Y, Haroon B, Bishop LD, Hawboldt J (2011). Antibiotic administration longer than eight hours after triage and mortality of community-acquired pneumonia in patients with diabetes mellitus. Eur J Clin Microbiol Infect Dis.

[4] Fee C, Weber EJ, Maak CA, Bacchetti P (2007). Effect of emergency department crowding on time to antibiotics in patients admitted with community-acquired pneumonia. Ann Emerg Med.

[5] Singh H, Thomas EJ, Sittig DF, Wilson L, Espadas D, Khan MM, Petersen LA (2010). Notification of abnormal lab test results in an electronic medical record: do any safety concerns remain?. Am J Med.

[6] Eisenberg RL, Yamada K, Yam CS, Spirn PW, Kruskal JB (2010). Electronic messaging system for communicating important, but nonemergent, abnormal imaging results. Radiology.

[7] Simonetti A, Viasus D, Garcia-Vidal C, Adamuz J, Roset A, Manresa F, Dorca J (2012). Timing of antibiotic administration and outcomes of hospitalized patients with community-acquired and healthcare-associated pneumonia. Clin Microbiol Infect.

[8] Houck PM, Bratzler DW, Nsa W, Ma A, Bartlett JG (2004). Timing of antibiotic administration and outcomes for Medicare patients hospitalized with community-acquired pneumonia. Arch Intern Med.

[9] Singh H, Arora HS, Vij MS, Rao R, Khan MM, Petersen LA (2007). Communication outcomes of critical imaging results in a computerized notification system. J Am Med Inform Assoc.

[10] Abujudeh HH, Kaewlai R, Choy G, Whelton DG, Rosenthal DI (2009). Important imaging finding e-mail alert system: experience after 3 years of implementation. Radiology.

[11] Horii S, Redfern R, Feingold E, Kundel H, Nodine C, Arnold D, Abbuhl S (2001). An automated results notification system for PACS. J Digit Imaging.

